# Emergency Ambulance Healthcare for People With Intellectual Disabilities: A Scoping Review

**DOI:** 10.1111/jar.70293

**Published:** 2026-08-18

**Authors:** Billy Ridler, Julia Sterman, Jennifer Murray, Valerie Houghton, Natasha Spassiani

**Affiliations:** ^1^ Edinburgh Napier University Edinburgh UK; ^2^ Australian Catholic University Fitzroy Victoria Australia; ^3^ Centre for Addiction and Mental Health Toronto Ontario Canada; ^4^ Department of Psychiatry University of Toronto Toronto Ontario Canada

## Abstract

**Background:**

People with intellectual disabilities experience a range of barriers when accessing healthcare. Ambulance services are uniquely placed within the healthcare system. Ambulance services are evolving to meet their full potential, with an increased focus on promoting health and tackling health inequalities. In this context it is timely to consider what is the experience of people with intellectual disabilities in relation to emergency ambulance healthcare.

**Method:**

Scoping review methodology was used to understand the experience of people with intellectual disabilities in relation to emergency ambulance healthcare.

**Results:**

Ten records were included in the review. Four core themes were identified: empathy, education, interprofessional working, and capacity and decision making. These themes represent enablers and barriers present for people with intellectual disabilities when accessing emergency ambulance healthcare.

**Conclusions:**

The scoping review has identified a significant gap in the literature, with the experience of people with intellectual disabilities being unknown.

## Lay Summary

- The experience of people with intellectual disabilities in relation to emergency ambulance healthcare is not known in published research or in grey literature.
- People with intellectual disabilities may experience barriers and enablers in relation to emergency ambulance healthcare.
- These barriers and enablers can be discussed across themes of empathy, education, interprofessional working, and capacity and decision‐making.
- Empirical research is needed to explicitly understand the experience of people with intellectual disabilities.

## 1 Introduction and Background

People with intellectual disabilities experience poorer health across a range of health indicators including self‐rated health, morbidity, cancer, diabetes, obesity, and polypharmacy (Emerson et al. 2016). Additionally, people with intellectual disabilities experience more co‐morbidities and long‐term health conditions than people without disabilities (Heslop et al. 2014). Due to these co‐occurring conditions people with intellectual disabilities come into contact with the healthcare system, including primary, secondary and emergency care, more frequently than non‐disabled people (Blaskowitz et al. 2019; Spassiani et al. 2020; York et al. 2022). When they engage with the healthcare system, people with intellectual disabilities experience discrimination, with their intellectual disabilities diagnosis overshadowing presenting health concerns, resulting in illnesses or disease not being appropriately diagnosed or treated (York et al. 2022). This discrimination contributes to people with intellectual disabilities experiencing a range of barriers across all contexts of the healthcare system including difficulties in communicating with healthcare providers, lower quality of care, disability‐related stigma, and a lack of respect from healthcare providers (United Nationals (UN) 2006; Spassiani et al. 2020). Barriers and facilitators reported by people with intellectual disabilities and/or autism, and their carers to accessing primary care included training, knowledge, communication, fear, involvement in decision making, and time (Doherty et al. 2020). These experiences infringe on the rights of people with intellectual disabilities. People with disabilities, including intellectual disabilities, have the right to the highest achievable standard of health and wellbeing without discrimination associated with their disability (United Nations 2006). The United Nations Convention on the Rights of Persons with Disabilities requires care, delivered by health professions to be of the same quality to persons with disabilities as to others; this convention has been adopted by the United Kingdom being presented to the United Kingdom Parliament in 2010 (Crown 2010). Despite this there is evidence that the rights of people with intellectual disabilities in relation to health and wellbeing continues to be infringed. Annual reporting, undertaken in England, shows that the rate of avoidable deaths for adults with intellectual disabilities who have died was 40.2% in 2023; this is compared to 21.8% in the general population (White et al. 2026).

A recent United Kingdom House of Commons Committee report concluded that there was a lack of appropriate training, awareness and specialist practitioners, such as learning (intellectual) disability nurses across the healthcare system and these factors contribute significantly to the health inequalities experienced by people with intellectual disabilities (Women and Equalities Committee 2024). The barriers identified in the report are mirrored within the Health Services Safety Investigations Body report that reviewed the care of people with intellectual disabilities within National Health Service acute hospitals within England. A lack of appropriate training, time and inconsistent implementation of specialist services such as learning disability liaison teams were all aspects identified as barriers to people with intellectual disabilities receiving safe care within acute settings (Health Services Safety Investigations Body 2023). The United Kingdom context, with a special focus on Scotland, is of particular interest for the authors due to the increased focus and initiatives being undertaken to promote the health and wellbeing of people with intellectual disabilities and address the health inequalities experienced (Scottish Government 2021; Scottish Government 2022). Additionally, the authors are undertaking a participatory action research study, in Scotland, that aims to collaboratively develop person‐centred emergency ambulance healthcare for people with intellectual disabilities.

Contact with healthcare systems, both acute and primary, may result from initial contact with an ambulance service. Ambulance services provide a combination of emergency, urgent unscheduled care as well as providing scheduled care such as supporting people to attend hospital appointments and facilitating admission and discharge from hospital (NHS England 2015; NHS England 2020; Scottish Ambulance Service (SAS)(b), n.d.). Within the United Kingdom, there has been an increased focus on the role of ambulance services when delivering emergency care, the systems and processes that connect ambulance services to the wider healthcare system, and patient journeys, experiences and outcomes following contact with an emergency ambulance (NHS England 2015). This focus has supported ambulance services, and their partners across healthcare systems, to introduce adaptive clinical models, providing increased opportunities for ambulance services to evolve from a transport to a treatment role. Contemporary ambulance healthcare now has an increased focus on avoiding unnecessary conveyance to acute environments. This shift is supported by increased use of remote assessment and treatment services, enhanced on scene assessment and treatment options and enhanced pathways to primary and scheduled care services (Mason 2022; NHS England 2015; Eaton 2023).

The contemporary role of ambulance services, and their clinicians, are reflected at a strategic level within services. The Scottish Ambulance Service state in their 2030 strategy that their vision is “saving more lives, reducing inequalities, improving health and wellbeing” (SAS(c), n.d.). Taking actions to tackle inequality, using a human rights‐based approach and planning, designing, and delivering services around people, and recognising the importance of peoples' lived experience are identified as approaches to achieving their vision (SAS(c), n.d.). These ambitions are not unique to ambulance services. There is an increasing emphasis on adopting rights‐based approaches to healthcare, where systems are underpinned by co‐production, shared decision making and tackling inequality. Healthcare systems, and providers, working to improve health and wellbeing for communities and individuals and not merely treating ill health underpins national contemporary healthcare policy and priorities (Public Health Scotland 2022).

Utilising approaches that promote human rights and person‐centred care has the potential to meet the health needs of people with intellectual disabilities more effectively than care models based on paternalism and limited, or no, shared decision making (Fenney et al. 2022; Shady et al. 2022). Despite this potential and the emphasis in contemporary policy on adopting these approaches the health inequality and barriers to receiving safe, effective, care continues to be consistently evidenced. This is not a new challenge. A 2014 systematic review of the experience of people with intellectual disabilities in hospital concluded that despite 20 years of research and government initiatives people with intellectual disabilities continued to have poor experiences in hospital (Lacono et al. 2014). This initial work has been built upon to provide an understanding of specific barriers that exist in the hospital context as well as primary care (Doherty et al. 2020; Health Services Safety Investigations Body 2023; Women and Equality Committee 2024).

The barriers experienced by people with intellectual disabilities appear to be known within acute and primary care, but within the reports, synthesis of evidence, and reviews, it is not clear if the experience of people with intellectual disabilities, when in contact with emergency ambulance services is known. It is important not to assume that the experience and barriers would be transferable among the different contexts. Ambulance services are uniquely positioned within healthcare systems as they respond to and deliver care in a range of different environments within the community. They are the only part of the system with responsibility to respond to people experiencing life‐threatening ill health in their own home/community and as a service are directly accessible to the public without the need for referral. The clinical models, approaches and tools utilised have been developed to support treatment in this context and care pathways include contact with primary and acute healthcare. The experience of people with intellectual disabilities in this context is important to understand as it may identify additional barriers and enablers for people with intellectual disabilities when receiving healthcare and support a wider understanding of the role ambulance services have in tackling or compounding the health inequalities experienced.

This review, which utilises scoping review methodology, aims to understand the experience of people with intellectual disabilities when accessing emergency ambulance healthcare. A secondary aim is to identify barriers and enablers to receiving and delivering care for people with intellectual disabilities. Furthermore, scoping review methodology allows for the identification of key concepts and terms associated with this area to support in identifying research gaps.

Research questions to support achieving these aims are: what is the experience of adults with intellectual disabilities when accessing emergency ambulance healthcare? Does this experience identify any barriers or enablers to safe, effective care and treatment? Are there gaps in the literature that prevent experience and barriers or enablers from being understood?

## 2 Methods

### 2.1 Development of the Search Strategy

This scoping review followed the Arksey and O'Malley (2005) and Joanna Briggs Institute methodologies (Peters et al. 2020). Additionally, the Preferred Reporting Items for Systematic reviews and Meta‐Analyses extension for Scoping Reviews (PRISMA‐ScR) was utilised to inform the structure and components of the review (Tricco et al. 2018). A scoping review protocol was developed by the authors and used to undertake the review. The protocol was not published but is accessible from the authors. Table 1 shares the participants, concept and context framework used to inform the initial steps within the review and identifies the eligibility criteria applied. Development of the proposed search strategy, including identification of final eligibility criteria, was an iterative process with a three‐step approach to identify published academic literature being taken, as recommended by Peters et al. (2020). Initial searches of Medline and CINAHL were undertaken with results from this search analysed for key words, phrases, language, and indexing to describe and identify relevant literature. Key words to inform these initial searches were identified in line with the population, concept and context framework.

**TABLE 1:** Inclusion and exclusion criteria.

	Inclusion	Exclusion
Participants	Adults with intellectual disabilities Carers of people with intellectual disabilities	Neurodivergent people without intellectual disabilities (autistic people, people with learning difficulties) People with acquired brain injuries
Concept	Statutory providers of ambulance care Emergency ambulance healthcare, typically provided in a statutory context and accessed via an emergency triage system All clinical response models utilised within ambulance healthcare that is, hear, treat, refer and/or see, treat, refer and/or conveyance to hospital Experiences of being in direct receipt of care, support and/or treatment from an ambulance provider/clinician Perceptions/beliefs/understanding of ambulance healthcare without direct experience Policy, strategy and practice guidance with regards to ambulance healthcare	Private providers of ambulance healthcare. Care/contact with ambulance clinicians not employed within an ambulance Service/Trust that is, a paramedic practitioner working in primary care or an emergency care setting
Context	International (for peer‐reviewed academic sources, not applicable to grey literature) Published in English	

Language used to discuss and describe disabilities—and people with disabilities—varies, and has changed over time (Andrews et al. 2022). Person first language is described as inclusive and reflected in contemporary guidance on use of language for authors (United Nations, n.d.). It is recognised that language preferences are nuanced, complex and vary for users and groups (Grech et al. 2024). This review utilises person first language and has adopted a definition of intellectual disability that is based on the presence of three criteria: intellectual impairment, social or adaptive dysfunctioning and early onset, typically in childhood (Holland 2011). Consistency of language in relation to disabilities within academic scholarship has evolved to reflect social and cultural changes, and the language preferences of people with disabilities (Andrews et al. 2022). In the United Kingdom the term learning disability is preferred over intellectual disability and is utilised by advocacy and representative groups (Mencap 2024). This article is intended for an international audience and as such the term intellectual disability, rather than learning disability is used. The term learning difficulty, which can be confused with learning/intellectual difficulties and at times preferred by people with disabilities (Holland 2011; Mencap 2024) typically relates to conditions such as dyslexia. These conditions do not meet the definition of intellectual disabilities adopted within this review so were excluded, as reflected in Table 1. People first language, is identified as inclusive and used within this article. The authors recognise that person first language is not preferred universally but is typically expressed as the preferred language by groups representing people with intellectual disabilities and internationally recognised health organisations (United Nations, n.d.; *Mencap*, n.d.; Grech et al. 2024). Terms and language which are now understood as offensive, and not appropriate, were included as key search terms purposefully with the aim of identifying historical sources and ensuring the search was robust and identified all relevant sources. The search was not limited by a date range, so inclusion of historical terms ensured sources published prior to evolution of language were identified. The authors recognise this language is not appropriate and do not endorse its use in contemporary literature.

Consistent with an iterative process all source types were searched simultaneously during this phase, and the search strategy was developed and refined prior to being finalised (Levac et al. 2010). Table 2 details the key search terms used.

**TABLE 2:** Search terms.

Adults with intellectual disabilities	Emergency ambulance healthcare
Intellectual disability, learning disability, developmental disability, mental retardation, down syndrome, William syndrome, fragile X syndrome, global developmental delay, autism, autism spectrum disorder	Ambulance, emergency care, emergency service, emergency medical service, out of hospital, prehospital, paramedic, technician, emergency medical technician, EMT, ambulance care, 999

The first author systematically searched four databases (Medline, CINAHL, PubMed and Science Direct) and hand searched six journals focused on emergency healthcare or intellectual disability (Journal of Intellectual Disability Research, Journal of Applied Research in Intellectual Disabilities, BMC Emergency Medicine, Journal of Paramedic Practice, British Paramedic Journal and Prehospital Emergency Care).

The grey literature search supported addressing the secondary research question due to the developing nature of ambulance services and the evolving nature of education for clinicians. Grey literature has the potential to inform practice and support making recommendations for practice (Adams et al. 2017). The grey literature search sought to identify practice priorities, themes and activity in United Kingdom‐based ambulance services in relation to intellectual disabilities. Additionally, it sought to identify the experience, views and perspectives of people with intellectual disabilities through self‐advocacy groups or representative organisations, with the potential that information appropriate may have been shared in blog posts, news articles or similar sources not routinely published in academic journals. The grey literature search was limited to the United Kingdom as the scoping review forms part of a wider research project that aims, through participatory action research, to understand and enhance ambulance healthcare for people with intellectual disabilities. Furthermore, the United Kingdom has a statutory model of provision for emergency ambulance healthcare, with ambulance services being health boards, or trusts, and not private—which is different from some international ambulance services. The authors did consider the inclusion of grey literature databases in the search strategy but concluded that inclusion was not aligned to the aims of the grey literature search. Appendix S1 details the grey literature search.

The literature searches, including empirical sources and grey literature initially took place in early 2024, with two authors refining the search strategy based on a sample of the initial results. Following peer review the search strategy was amended, and the searches undertaken again in June 2026. The databases were searched from inception, with no date limitations utilised. Date limitations were not utilised as the review hoped to identify all relevant sources in this previously unexplored subject area. Appendix S2 presents the full search strategy undertaken in Medline.

### 2.2 Screening and Selection of Sources

**FIGURE 1:**
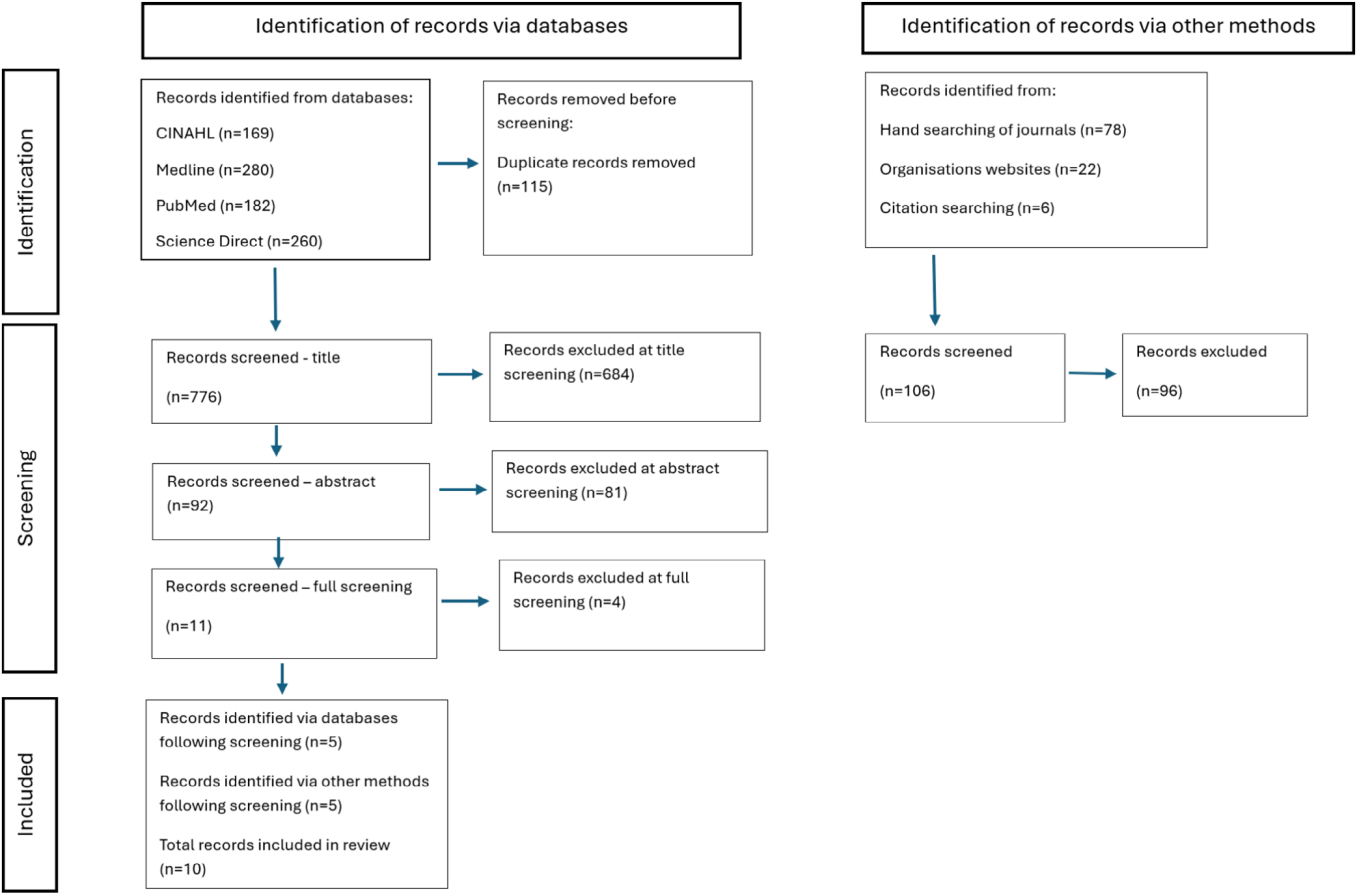
PRISMA 2020 flow diagram (Tricco et al. 2018).

Results were exported into a reference management system and duplicate records were removed. Remaining sources were screened by title and abstract. Next, full texts for sources were screened to determine eligibility for inclusion. Grey literature sources were screened in full for inclusion. Additionally, reverse citation searching was undertaken for the peer‐reviewed sources identified for inclusion; this identified six further sources for review. Forward citation was undertaken for a sample of the peer‐reviewed sources included. Due to forward searching not yielding any additional sources that met the inclusion criteria, the authors decided to pursue additional forward citation searching. Screening was undertaken by the primary author, as this scoping review has been completed in the context of a doctoral studentship. Two authors worked on the development of the search strategy, reviewed a sample of the initial results to ensure appropriateness and screened for potential eligibility. Co‐authors were available to discuss sources where the primary author had uncertainty on inclusion or exclusion. A consensus on inclusion would have then been taken. This was not required, with the clear inclusion and exclusion criteria and limited range of sources identified being features that enabled effective screening. Figure 1 provides a PRISMA flow diagram (Tricco et al. 2018) to illustrate the records identified, and excluded or included, at each stage.

### 2.3 Data Extraction and Analysis

Consistent with scoping reviews, the focus of the analysis was to collate, summarise, and report results of included sources (Arksey and O'Malley 2005; Sterman and Njelesani 2021). Data extraction was undertaken by the primary author using a data extraction form. A data extraction form was developed informed by the principles of Pollock et al. (2023), with the aim of extracting key information from the sources to facilitate summarising the results of the review in a logical and descriptive manner. As recommended for scoping reviews (Arksey and O'Malley 2005; Peters et al. 2020), the extraction form was piloted with a sample of sources. Following the pilot, amendments were made to the data extraction tool to aid in capturing, more specifically, the key messages, findings and content pertaining to the aims of the review. Data extraction was undertaken by the primary author, as the scoping review forms part of their doctoral studies. The final version extracted title, author(s), year of publication, associated organisation(s), source type, aim/purpose of the source, study design, participants/population, intervention, results, main findings, recommendations and relevance of the source to the objectives of the scoping review. All information that was extracted informed the review. Additionally, an adapted exploratory thematic analysis approach was taken to synthesise the sources and identify core themes across them (Arksey and O'Malley 2005; Sterman and Njelesani 2021). This approach enabled new knowledge to be generated in this area.

## 3 Results

The full screening process identified 10 sources eligible for inclusion within the review. These 10 sources consisted of one ambulance trust strategy document, a mixed methods exploratory‐descriptive study, five cross‐sectional studies, a longitudinal study, a reflective account, and a continued professional development module. Publication dates range from 2012 to 2022. The sources represent publications from the United Kingdom (*n* = 3), Canada (*n* = 2), the United States of America (*n* = 1), and Australia (*n* = 4). Table 3 details the characteristics of the included sources. Seven of the sources represent empirical research, with paramedic students and emergency medical service providers representing the populations sampled within the sources (Williams et al. 2012; Williams et al. 2013; Williams et al. 2015a; Williams et al. 2015b; Kus et al. 2018; Pagano et al. 2018; McGinley et al. 2017). The other sources represent experiences of United Kingdom‐based paramedic students and learning disability nursing students and expression of professional knowledge or expertise from educators, academics, a clinical academic and a learning (intellectual) disability and vulnerabilities specialist (Harper et al. 2019; Moritz et al. 2020; Howe 2022). One source involved a stakeholder feedback event to inform its development, with representatives from United Kingdom‐based learning disability health and social care teams, learning disability commissioning leads, general practice leads and acute learning disability liaison nurses (Howe 2022). No studies identified represent populations of people with intellectual disabilities. Table 3 provides an overview of the included sources.

**TABLE 3:** Features of the sources included in the review.

Source (Location)	Source type	Source Aim	Population	Findings	Recommendations
Moritz et al. (2020). (United Kingdom)	Published continued professional development module/resource	Provision of professional development in relation to vulnerability, identification of vulnerable groups, discussing autonomy and capacity in vulnerable groups and reflection on own practice in relation to protecting vulnerable groups	Not applicable	Not applicable	Not applicable
Harper et al. (2019) (United Kingdom)	Published Reflection	Reflection on an inter‐professional simulation event for paramedic and learning disability nursing students	Not applicable	Not applicable	Not applicable
Pagano et al. (2018) (Canada)	Longitudinal study	Study aimed to determine the empathy levels demonstrated by first year paramedic students over the course of their first year of study	First year paramedic students in Canada (*n* = 20)	Empathy levels overall declined over time Male paramedic students were less empathetic than female counterparts Paramedic students with previous post‐secondary education had higher mean empathy scores	Further research of larger sample sizes, in different regions and measuring empathy levels towards other medical conditions Research to understand the levels of empathy in qualified paramedics in clinical environments Paramedic education programmes should consider implementing greater empathy training and education
Kus et al. (2018) (Canada)	Cross sectional study	Study aimed to assess empathy scores of first and second d year paramedic students in Canada	Paramedic students (*n* = 43)	Physical disability and intellectual disability were held in similar regard Substance abuse was held in significantly lower regard Age does not play a significant role in influencing empathy. First year students had a higher mean empathy score compared with second year students Female students displayed higher mean empathy scores	Further research that is longitudinal in nature among paramedic students. Research exploring empathy levels among qualified paramedics, longitudinally, to understand empathy levels over a career Further research exploring reasons for changes throughout a career Research to determine required action within education of paramedic students to improve empathy levels and how best to do this
McGinley et al. (2017). (USA)	Exploratory‐descriptive study	Study aimed to describe how medical orders inform Emergency Medical Service (EMS) providers' decision‐making during emergencies involving people with intellectual disabilities who are near life's end	EMS providers (*n* = 239)	62.7% of the sample had treated a person with intellectual disabilities experiencing end of life care Provider familiarity, organisational processes and sociocultural context were three themes identified as influencing how medical orders informed EMS providers during these episodes of care	Further research exploring end of life wishes and care for people with intellectual disabilities should be situated within a sociocultural context Further research is required to understand the process of advance care planning among surrogates, the certification of medical orders and the congruency of documented wishes
Howe (2022) (United Kingdom)	Ambulance Trust Strategy	Expresses the vision the trust has to support people with intellectual disabilities who access the Trusts services.	Not Applicable	Not Applicable	Not Applicable
Williams et al. (2015a) (Australia)	Prospective cross‐sectional longitudinal study	Study aimed to examine the attitudes of undergraduate paramedic student towards patients with intellectual disabilities, substance abuse, acute mental illness and those who attempted suicide.	Undergraduate paramedic or paramedic/nursing students (*n* = 554)	Single degree students held a higher regard for people with intellectual disabilities No significant change for single‐degree students over their course Double degree students showed a positive change towards people with intellectual disabilities, substance abuse and attempted suicide over their course People presenting with substance use were held in lower regards in comparison to the other patient populations Female students, generally, had a higher regard for people with overdose, substance abuse and attempted suicide compared to males	None identified
Williams et al. (2015b) (Australia)	Cross sectional study	Study aimed to determine the attitudes of first year paramedic students entering their course in year one over four consecutive yearly intakes towards patients with intellectual disabilities, substance abuse, attempted suicide and acute mental illness	First year paramedic and or paramedic/nursing course students (*n* = 230)	Participants had a relatively poor attitude towards the four patient populations Participants showed the most negative attitudes towards people with substance abuse They held the most positive attitude towards people with intellectual disabilities No significant difference between genders and age groups	Further research involving all year levels across multiple sites/universities Qualitative analysis and content review to understand the relationship between results and curricula Targeted educational curricula supporting empathy awareness in stigmatised patient populations Research exploring the impact of heightened awareness on career longevity and work satisfaction
Williams et al. (2013). (Australia)	Cross sectional study.	Study aim was to assess the extent of empathy in paramedic students enrolled at a particular University.	Paramedic students (*n* = 94)	Male students had higher mean empathy scores, when compared with female students Empathy scores were lowest among the first‐year students No significant difference between age groups was identified Substance abuse scored the lowest levels of compassion First and second year students had significantly more regard for intellectual disabilities, attempted suicide and acute mental illness when compared with third year students	Future testing should incorporate a general medical condition or a specific psychiatric condition within the measurement instrument
Williams et al. (2012). (Australia)	A cross‐sectional study	The study aimed to assess the extent of empathy in paramedic students across seven universities.	Paramedic students (*n* = 783)	Across intellectual disabilities, attempted suicide and acute mental illness mean scores were above 50 suggesting good empathetic regard People presenting with substance abuse produced the lowest mean score from students Female students report higher levels of empathy than male counterparts across each patient population, with the difference being statistically significant for people with intellectual disabilities	Longitudinal studies by research teams Educators to begin developing guidelines focusing on incorporating, promoting and instilling empathy into paramedic students

The review was unable to address the primary question, as none of the sources identified for inclusion represented perspectives of people with intellectual disabilities, meaning the experience of people with intellectual disabilities remains unknown in the emergency ambulance context. Thematic analysis identified four core themes across the sources: empathy, education, interprofessional working, and capacity and decision‐making. These themes represent enablers and barriers, addressing the secondary research question.

## 4 Empathy

Empathy was identified as a major theme in the sources, with six of the sources explicitly researching empathy levels in paramedic students towards a range of populations, including people with intellectual disabilities (Williams et al. 2012; Williams et al. 2013; Williams et al. 2015a; Williams et al. 2015b; Kus et al. 2018; Pagano et al. 2018). One of the sources also included students undertaking a dual nursing and paramedicine programme (Williams et al. 2015a). However, four of these sources share the same primary author. The volume of publication by this author and focus of their research may have contributed to an inflation of the theme of empathy being represented within the sources (Williams et al. 2012; Williams et al. 2013; Williams et al. 2015a; Williams et al. 2015b). Most of the sources use a cross‐sectional design, three of the studies are longitudinal in nature. Four of the sources recruit samples from Australian higher education institutions, the other two sources recruit from higher educational institutions in Canada (Williams et al. 2012; Williams et al. 2013; Williams et al. 2015a; Williams et al. 2015b; Kus et al. 2018; Pagano et al. 2018). Consistent across all sources is the use of the Medical Condition Regard Scale to capture empathy levels and attitudes. The Medical Condition Regard Scale is a valid tool for examining, and assessing, regard for medical conditions and through the 18‐item scale captures emotions, expectations, and biases through medical condition descriptors (Christison et al. 2002). Within the sources frequently people with intellectual disabilities, substance abuse, acute mental ill health and suicide were the patient populations explored. Levels of empathy and attitude within paramedic students appears mixed, with one source reporting relatively poor attitudes towards all the populations explored (Williams et al. 2015b). In contract Williams et al. (2012) reports mean scores reflective of good empathetic regard across people with intellectual disabilities, attempted suicide, and acute mental illness. Consistent across the sources is that attitudes towards substance abuse are more critical, with lower levels of empathy among the samples. People with intellectual disabilities, in comparison, attract higher levels of empathy, with Williams et al. (2015b) concluding their sample held the most positive attitude towards people with intellectual disabilities when compared to the other populations. Despite people with intellectual disabilities garnering relatively high empathy, overall, empathy levels were low, even in first year students, (Williams et al. 2013) and typically decline over an ambulance clinician's career (Pagano et al. 2018; Kus et al. 2018; Williams et al. 2013). Some of the sources found that female students held higher levels of regard, when compared to male students (Williams et al. 2012; Williams et al. 2015a; Kus et al. 2018; Pagano et al. 2018). Williams et al. (2012) found a statistically significant difference in levels of regard for people with intellectual disabilities between male and female students. This is not evidenced across all the sources with Williams et al. (2015b) finding no difference between genders and Williams et al. (2013) finding higher mean empathy in male students. Williams et al. (2012) in addition to gender, found statistical differences across age groups and educational institutions attended by paramedic students. The study sampled students across seven universities and found differences in levels of regard across them. These differences highlight that paramedic students cannot be regarded as one group, with consistent levels of empathy. Age, higher educational institutions attended, gender and stage of study appear to be influencing factors, although not all of these factors can be evidenced as statistically significant or influencing levels of empathy consistently.

## 5 Education

Education is represented across a range of the sources and represents a core theme. The ambulance trust strategy discusses two aspects of education; education for employees within the trust and education for the community who access their care. The strategy makes commitments to connect with communities to teach and share information on what to do in an emergency and ways to contact the service when help is required (Howe 2022). They describe a programme of work that aims to bring awareness to demystify the ambulance service and reduce anxiety for people with intellectual disabilities when in contact with the service. They plan to produce easy read and digital resources for people with intellectual disabilities to express this information and the educational messages (Howe 2022). Education for employees of the trust includes the implementation of statutory and mandatory training for all, with the education required for each role being mapped to an appropriate level of a national core capabilities framework. This framework is focused on working with people with intellectual disabilities (Skills for Health et al. 2019). It is anticipated education will focus on signs of deterioration, equality legislation and making reasonable adjustments, communication and assessing capacity. The opportunity to engage in this learning when practising as a paramedic may be an enabler to enhanced emergency ambulance healthcare as a source identified within the review offers a reflection from a paramedic student that they have limited experience with patients with intellectual disabilities (Harper et al. 2019). However, this is a single student's reflection. Educational approaches that involve people with intellectual disabilities may act an enabler to enhanced care as feedback from a person with intellectual disabilities during a simulated scenario was described as improving a paramedic student's confidence (Harper et al. 2019).

## 6 Interprofessional Working

This review has identified limited evidence explicitly exploring paramedic students, or registered paramedics, theoretical or practice education in relation to intellectual disabilities. One source offers reflections on an inter‐professional simulation event for paramedic and learning disability nursing students (Harper et al. 2019). This source contributes to the core themes of education and interprofessional working as the objective of the event was to increase understanding, across the attendees, of each other's roles and how they can work together, as services, to deliver coordinated, collaborative care for a person with intellectual disabilities. Four participants offered reflections sharing how they gained an increased understanding of one another's knowledge, scope of practice and core competencies and how this increased understanding would enable them better to seek support from one another. It was also highlighted by the paramedic students that, through the experience, they identified the importance of adaptive approaches to communication and consultation, and the nursing students felt they could offer guidance in relation to this (Harper et al. 2019). Howe (2022) describes an ambition to improve interprofessional working with a focus on joint working and multi‐agency communication at an organisational level. Additionally, limited consistent and clear pathways of care are identified as a barrier to optimal care for people with intellectual disabilities (Howe 2022). Complex scenarios can give rise to situations where ambulance services are looking for input from a range of other professionals and teams to meet the person's needs. A barrier to accessing this consistently is the availability of primary and specialist community healthcare teams which is largely 9 am‐5 pm Monday–Friday, whereas ambulance services operate continuously. This means that during unscheduled hours ambulance clinicians have difficulty in accessing other professionals, or services, that know the person or who can meet the person's needs (Howe 2022). The strategy document is not peer reviewed or empirical in nature but does reflect stakeholder feedback including specialist learning disability health and social care teams and ambulance service staff.

## 7 Capacity and Decision Making

The final theme identified within the sources was capacity and decision making. One of the sources is a continued professional development resource focussed on ethics, capacity, and vulnerable patients (Moritz et al. 2020). The resource focussed on English capacity legislation discusses the role of paramedics in supporting and responding to a range of groups including children, older people, people experiencing mental illness and disabled people, including people with intellectual disabilities. It identifies that people with intellectual disabilities may lack capacity and require support from carers to assist in decision making that aligns with their values, while other carers may have full responsibility for decision‐making (Moritz et al. 2020). This is a potential challenge for clinicians when they are seeking to consider the individual's preferences and wishes if another person is responsible for their decision‐making. McGinley et al. (2017) also reflect a concern from emergency medical service providers that, when decisions and choices are being made on behalf of a person with intellectual disabilities, they may not fully represent what the person would want. Both sources discuss the availability of written information such as advanced care planning documents or tools as being enablers to providing care, as often they have been completed with family and health and social care professionals and articulate the preferences and wishes of the personA with intellectual disabilities (McGinley et al. 2017; Moritz et al. 2020). The individual nature of autonomy, the changing nature of capacity depending on multiple circumstances and the potential unconscious biases of paramedics towards disabled people are also all identified as potential barriers to optimal care and shared decision making (Moritz, 2020).

## 8 Discussion

The scoping review sought to understand the experience of adults with intellectual disabilities when accessing emergency ambulance healthcare. The sources identified have been unable to provide this understanding. The review identified no representative studies that have sought to understand the perspective, experience, or utilisation of emergency ambulance healthcare by people with intellectual disabilities. This contrasts with other areas of emergency healthcare. York et al. (2022) identified 14 studies exploring people with intellectual disabilities and emergency care with these studies representing international research across hospital emergency departments, inpatient hospital admissions, urgent primary care with subsequent hospital admission, hospital use and ambulatory clinics utilisation. This scoping review did identify core themes across the sources included and these themes describe some potential enablers and barriers to emergency ambulance healthcare for people with intellectual disabilities.

Within the sources empathy is a significant theme; however the number of publications from one primary author likely contributed to the prominent representation of this theme among the sources (Williams et al. 2012; Williams et al. 2013; Williams et al. 2015a; Williams et al. 2015b). Empathy, within healthcare, is understood as the professional's ability to understand the feelings, perspective, reactions, and the experience of others (Moudatsou et al. 2020; Kus et al. 2019). The sources within the review suggest that levels of empathy within paramedic students are variable. Empathy, as a key tool in the formation of health professional‐patient relationships, has been evidenced as supporting enhanced patient outcomes, being key to patient experiences and a critical component of person‐centred care (Moudatsou et al. 2020; Nembhard et al. 2023). Empathy is effective, within general practice, in lowering anxiety for patients, reducing distress, strengthening enablement, and enhancing clinical outcomes (Derksen et al. 2013). Evidence exploring the impact and role of empathy has been developed across nursing, medicine and other health and social care disciplines but less so in paramedic practice, with empathy in paramedic practice being understudied (Kus et al. 2019). It may be possible that enhanced levels of empathy within emergency ambulance healthcare could enhance experiences and outcomes for people. It is unclear how empathy levels translate to the experience or outcomes of people accessing emergency ambulance healthcare. It may be hypothesized, given the evidence in other healthcare contexts, that higher levels of empathy in emergency ambulance clinicians would support better care; this has not been empirically studied, as it has in other settings (Derksen et al. 2013; Kus et al. 2019).

The review does not make it clear what experience, understanding, or training emergency healthcare clinicians have regarding empathy. However, studies examining empathy levels among paramedic students suggest that educators and paramedic programmes should develop additional, targeted education and training on empathy within their curricula (Pagano et al. 2018; Kus et al. 2018; Williams et al. 2015b; and Williams et al. 2012). They recommend these amendments with the aim of heightening levels of empathy towards stigmatised patient populations. It is hoped this education will contribute to a level of care that is standard, across the different patient populations, and facilitate positive relationships with patients (Kus et al. 2018). Education that focuses on enhancing empathy is effective in healthcare education (Winter et al. 2020). Winter et al. (2020) assessed the effect of empathy‐enhancing learning experiences within healthcare and found a range of interventions being used to enhance empathy. The most common learning experience was communication skill‐based training; additionally perspective‐taking training and broader empathy skill‐based training sessions were also utilised, alongside mixed learning experiences (Winter et al. 2020). The current educational standards and proficiencies in the United Kingdom for paramedic programmes make limited reference to empathy; there are no specific proficiencies reflecting skills or knowledge in relation to empathy (Health and Care Professions Council (HCPC) 2023). A subject benchmarking statement (The Quality Assurance Agency for Higher Education 2019) when describing the evolving contexts in which paramedics are practising discusses the importance of paramedics demonstrating empathy as key attribute, value, and behaviour in treating and caring for patients and supporting relatives and those with the patient. The paramedic curriculum guidance does state that paramedic students should “demonstrate an understanding of the needs of various service user groups, across the lifespan, including but not limited to…learning disabilities” (College of Paramedics 2019, pg. 17). The review identified no evidence on how education in relation to empathy has been integrated into paramedic programmes, or the impact of any such training for paramedics or people accessing care. However enhanced practice in relation to empathy may support the enhanced patient experience, patient‐professional relationship and a reduction in distress, anxiety and strengthen enablement as evidenced in other areas of healthcare (Derksen et al. 2013). Additionally, when considering the impact on care for people with intellectual disabilities empathy is an approach that can be used by professionals to recognise people with intellectual disabilities and overcome the barriers of exclusion and others talking or acting on their behalf (Skarsaune 2023).

With empathy being a key attribute of person‐centred care (The Health Foundation 2016), it is interesting to consider how intellectual disabilities is positioned within the sources researching empathy included in the review (Williams et al. 2012; Williams et al. 2013; Williams et al. 2015a; Williams et al. 2015b; Kus et al. 2018; Pagano et al. 2018). By using the Medical Condition Regard Scale, they position intellectual disabilities as a medical condition. This positioning would be consistent with the medical model of disability. Within the medical model, disability is understood as residing within the individual. This perspective fails to acknowledge environmental factors, such as political, social or physical influences that may contribute to a person's experience of disability (Shyman 2016; Fenney et al. 2022). The model focuses on diagnosis and categorisation to determine appropriate treatment, assuming that because disability is located within the person, intervention must come from an external source to compensate for the individual's perceived deficits (Shyman 2016).

Positioning intellectual disabilities within the medical model contradicts key principles of person‐centred care, such as dignity, compassion, and respect, as well as coordinated, personalised, and strength‐based supports. Person‐centred care emphasises enabling individuals to recognise and build on their strengths and abilities to live fulfilling lives (The Health Foundation 2016). Conceptualising intellectual disabilities as a single medical condition could be a barrier to optimal person‐centred emergency ambulance healthcare as the model does not prioritise understanding the person as an individual, including their strengths, capabilities, and environment. Additionally, it does not enable co‐creation due to the power imbalance within the relationships. Person‐centred care and co‐creation are associated with increased care satisfaction, improved physical and social well‐being, especially in those with long‐term and multiple health conditions, and it may lead to enhanced health outcomes for people (Kuipers et al. 2019; McMillan et al. 2013). Interestingly, Meer et al. (2018) evidenced that providing person‐centred and co‐created care for people with intellectual disabilities may support enhanced well‐being and job satisfaction for professionals working with people with intellectual disabilities. It follows that failing to position intellectual disabilities within a person‐centred model that facilitates shared decision‐making and co‐creation within emergency ambulance healthcare may result in poorer experiences, decreased satisfaction and a poorer experience for clinicians.

It is unclear the extent to which person‐centred care, as a model, has been adopted or introduced to emergency ambulance healthcare. A study of ambulance clinicians' experiences of person‐centred care in Sweden failed to identify any ambulance services that had introduced person‐centred care as a complete framework (Rantala et al. 2019). Rantala et al. (2019) found that person‐centred care acted as both an enabler and barrier to care for the ambulance clinicians they interviewed. The participants described the nature of ambulance care being one patient, often in their own home, supported with them seeing the whole person and gaining an understanding of their context. However, it was recognised that the medical model was dominant within their organisation and evidenced through elements such as clinical guidelines, assessment and measurement tools and decision‐making support approaches that were all focused on medical issues (Rantala et al. 2019).

The Scottish Ambulance Service (c) (n.d.) 2030 strategic document refers to person‐centred care when describing their ambitions in mental health care and describes an ambition to underpin realistic medicine within all the care they provide. Realistic medicine principles encourage clinicians to identify what matters to patients, treat patients as equal partners in decision making and share decisions about treatment options so that patients can make decisions that are right for them (Scottish Ambulance Service (c), n.d.). The review evidenced that clinicians within emergency ambulance healthcare can find it challenging to identify the preferences or wishes of people with intellectual disabilities (McGinley et al. 2017 and Moritz et al. 2020). There is evidence that there are challenges and barriers to shared decision making in other healthcare contexts for people with intellectual disabilities also, despite the increasing emphasis and focus within health and social care on shared decision making (Noorlandt et al. 2020). These barriers to shared decision making infringe on the rights of people with intellectual disability in relation to health and wellbeing. Alliance (2024) describe people with intellectual disabilities as a population who experience increased marginalisation associated with inequality. Adoption of rights‐based decision‐making, and practices, within healthcare settings is one approach proposed to support in addressing aspects of the inequalities experienced with the aim of realising the rights individuals have in regard to health (Alliance 2024; Scottish Commission for People with Learning Disabilities 2025).

The lack of evidence explicitly representing the experience of adults with intellectual disabilities and their experiences of emergency ambulance healthcare may be due to dominance of the medical model within the sector and the historical context of ambulance services primarily being responsible for responding to critically ill people, and transporting them to a hospital (Eaton 2023). This is not reflective of contemporary ambulance healthcare, with ambulance services now positioned as mobile healthcare providers, in addition to emergency care providers (Eaton 2023; SAS (a), n.d.; NHS England 2020).

Future research should look to explore the experience of people with intellectual disabilities when accessing emergency ambulance healthcare using representative research approaches. Developing an understanding of when, how, and why people with intellectual disabilities access emergency ambulance healthcare may support in understanding the health needs of people with intellectual disabilities further. Additionally seeking to understand the enablers and barriers to accessing care may support ambulance services in developing optimal approaches to meeting the needs of people with intellectual disabilities.

## 9 Limitations

Limitations of this review include excluding non‐English language sources, due to the language limitations of the main authorship team and the grey literature search being limited to United Kingdom‐based ambulance services and organisations. Forward citation searching was undertaken on a sample of included sources, rather than all the sources. Despite the sample yielding no results, full forward citation searching may have identified additional sources to be considered for inclusion. All stages of screening for inclusion and exclusion were undertaken by the primary author, rather than two independent reviewers. The risk associated with this limitation was mitigated by co‐authors being available to discuss any sources where inclusion or exclusion was unclear. Data extraction and the thematic analysis was also undertaken by the primary author. A wider team may have represented a depth of discussion and scrutiny that produced different results. Finally, the sixth, optional phase, of consultation which is recommended within Arksey and O'Malley's (2005) methodological framework has not yet been undertaken. This stage is anticipated to take place in the autumn of 2026 with an in person consultation event bringing together people with intellectual disabilities, representative organisations and emergency ambulance care clinicians and leaders.

A limitation of the sources that explored the levels of empathy in paramedic students is that they appear to present the patient populations being explored as homogeneous groups. There is insufficient information to identify if the individual nature of people with intellectual disabilities and communities is expressed or understood by the participants. This limitation is applicable to many of the sources where people with intellectual disabilities are discussed in a collective, rather than individual, manner with limited acknowledgement that this approach implies homogeneity. This approach has been identified as a barrier to optimal care with Bebbington and Croot (2025) finding that recognising heterogeneity and person‐centred care are interdependent and essential for optimal end of life care for people with intellectual disabilities. Despite this, recognising diversity within populations is challenging in clinical practice (Linares‐de‐Marcos et al. 2026).

## 10 Conclusion

This scoping review sought to explore the experiences of people with intellectual disabilities when accessing emergency ambulance healthcare. However, the systematic approach identified no representative empirical research that explicitly examined the experience of people with intellectual disabilities in emergency ambulance healthcare. Sources were identified that met the inclusion criteria and from these sources a range of potential enablers and barriers to receiving or delivering optimal healthcare was identified. As a core theme empathy represented a broad range of associated concepts that may influence care for people with intellectual disabilities, including the nature of person‐centred care within emergency ambulance healthcare, the dominance of the medical model within the sector and limited focus on empathy within paramedic education standards, despite evidence that it can enhance levels of empathy and in turn the experience of people in receipt of care.

The scoping review has identified a significant gap in the literature, with the experience of people with intellectual disabilities in this uniquely positioned area of healthcare being unknown. This gap is important because without understanding the experiences of people with intellectual disabilities in emergency ambulance healthcare, it is difficult to ensure that services are equitable, accessible and responsive to their needs. Emergency situations often involve high stress, communication challenges, and rapid decision‐making, which can disproportionately disadvantage people with intellectual disabilities. Identifying and understanding their experiences is therefore essential for informing training, policy, and practice improvements that promote person‐centred, inclusive emergency care.

## Author Contributions

B.R. developed the initial scoping review protocol, and undertook the primary database and grey literature searches, reviewing of resources for inclusion/exclusion, data extraction and thematic analysis. Developed initial draft of manuscript and has led on the revisions from peer review cycles. J.S. provided review, and enhancement, of the scoping review protocol. With B.R. undertook database searches to identify optimal key terms and screened sample of initial results. Provided extensive review, and re‐drafting of manuscript during initial development and following peer feedback. J.M. provided review, and enhancement, of the scoping review protocol. Provided extensive review, and re‐drafting of initial manuscript. V.H. provided extensive review, and re‐drafting of manuscript during initial development and following peer feedback. N.S. over saw the project as senior academic supervising B.R., subject expert for participatory methodologies and intellectual disabilities and health and wellbeing. N.S. provided review, and enhancement, of the scoping review protocol. Provided extensive review, and re‐drafting of manuscript during initial development and following peer feedback.

## Funding

The corresponding author is the recipient of a funded doctoral programme through Edinburgh Napier University.

## Ethics Statement

The authors have nothing to report.

## Consent

The authors have nothing to report.

## Conflicts of Interest

The authors declare no conflicts of interest.

## Supporting information


**Data S1:** Supporting Information.
**Appendix S1:** Grey Literature Search Overview.
**Appendix S2:** Medline Search Strategy.

## Data Availability

Data sharing not applicable to this article as no datasets were generated or analysed during the current study.
