# Supplementary material for: Emergency Ambulance Healthcare for People With Intellectual Disabilities: A Scoping Review
Source: J Appl Res Intellect Disabil. 2026 Aug 18;39(4):e70293. doi: 10.1111/jar.70293 (PMC13484159; doi:10.1111/jar.70293)
Supplement: Supplementary file 1 [file JAR-39-e70293-s001.docx]

Online Supplementary Appendix 1. Grey Literature Search Overview

The homepage of each organisation's website was searched using a sub-set of key terms as identified below.

| Organisation | Sub-set of Key Terms |
| --- | --- |
| Association of Ambulance Chief Executives,  East of England Ambulance Service,  East Midlands Ambulance Service,  London Ambulance Service,  North East Ambulance Service,  North West Ambulance Service,  South Central Ambulance Service,  South East Coast Ambulance Service,  South Western Ambulance Service,  West Midlands Ambulance Service,  Yorkshire Ambulance Service,  Welsh Ambulance Service,  Northern Ireland Ambulance Service,  Scottish Ambulance Service. | Intellectual disability, intellectual disabilities, learning disability, learning disabilities, developmental disability, developmental disabilities, autism |
| Mencap,  Learning Disability England,  British Institute of Learning Disabilities,  Foundation for People with Learning Disabilities,  Scottish Commission for People with Learning Disabilities. | Ambulance, paramedic, emergency |

Online Supplementary Appendix 2. Medline Search Strategy

| Search intellectual disabilities | Search Terms | Results |
| --- | --- | --- |
| S1 | (MH “Intellectual Disability”) OR (MH “X-Linked Intellectual Disability”) OR (MH “Learning Disabilities) | 78,912 |
| S2 | (MH “Autistic Disorder”) OR (MH “Autism Spectrum Disorder”) | 51,587 |
| S3 | XB ( Intellectual* N2 disab* or learning N2 disab* or development* N2 disab* or mental* N2 retard* or down* syndrome or william* syndrome or fragile X syndrome or global developmental delay or autism or autism spectrum disorder ) | 181,685 |
| S4 | (MH “Ambulances”) | 7,502 |
| S5 | (MH “Paramedics”) | 459 |
| S6 | XB ( Ambulance* or emergency care* or emergency service* or emergency medical service* or “out of hospital” or prehospital* or pre*hospital* or pre#hospital* or paramedic* or technician* or emergency medical technician* or EMT or ambulance* care or 999) | 142,204 |
| S7 | S1 OR S2 OR S3 | 225,223 |
| S8 | S4 OR S5 OR S6 | 144,162 |
| S9 | S7 AND S8 | 280 |
